# Cigarette Smoke Stimulates SARS-CoV-2 Internalization by Activating AhR and Increasing ACE2 Expression in Human Gingival Epithelial Cells

**DOI:** 10.3390/ijms22147669

**Published:** 2021-07-18

**Authors:** Cassio Luiz Coutinho Almeida-da-Silva, Harmony Matshik Dakafay, Kaitlyn Liu, David M. Ojcius

**Affiliations:** 1 Department of Biomedical Sciences, Arthur A. Dugoni School of Dentistry, University of the Pacific, San Francisco, CA 94103, USA; hmatshikdakafay@pacific.edu; 2 Dental Surgery Program, Arthur A. Dugoni School of Dentistry, University of the Pacific, San Francisco, CA 94103, USA; k_liu13@u.pacific.edu

**Keywords:** cigarette smoke, smoking, ACE2, TMPRSS2, SARS-CoV-2, COVID-19, AhR

## Abstract

A large body of evidence shows the harmful effects of cigarette smoke to oral and systemic health. More recently, a link between smoking and susceptibility to coronavirus disease 2019 (COVID-19) was proposed. COVID-19 is due to infection with severe acute respiratory syndrome coronavirus 2 (SARS-CoV-2), which uses the receptor ACE2 and the protease TMPRSS2 for entry into host cells, thereby infecting cells of the respiratory tract and the oral cavity. Here, we examined the effects of cigarette smoke on the expression of SARS-CoV-2 receptors and infection in human gingival epithelial cells (GECs). We found that cigarette smoke condensates (CSC) upregulated ACE2 and TMPRSS2 expression in GECs, and that CSC activated aryl hydrocarbon receptor (AhR) signaling in the oral cells. ACE2 was known to mediate SARS-CoV-2 internalization, and we demonstrate that CSC treatment potentiated the internalization of SARS-CoV-2 pseudovirus in GECs in an AhR-dependent manner. AhR depletion using small interference RNA decreased SARS-CoV-2 pseudovirus internalization in CSC-treated GECs compared with control GECs. Our study reveals that cigarette smoke upregulates SARS-CoV-2 receptor expression and infection in oral cells. Understanding the mechanisms involved in SARS-CoV-2 infection in cells of the oral cavity may suggest therapeutic interventions for preventing viral infection and transmission.

## 1. Introduction

Smoking harms nearly every organ in the body, and remains the leading cause of preventable disease, disability, and death in the United States [1,2]. Even though the current smoking rates have been declining, in 2019 approximately 34.1 million Americans were currently smoking [3], especially in the Midwest and South of the United States. Tobacco smoke contains a mixture of deadly substances, including more than 7000 chemicals, hundreds of which are toxic and about 70 that are known cancer-causing agents [2]. Smoking has been linked to several diseases, such as cardiovascular disease [4,5], cancer [6,7,8], diabetes [9], and acute severe respiratory distress syndrome (ARDS) [10,11,12]. More recently, smoking has been linked to the current pandemic of coronavirus disease 2019 (COVID-19) [13].

The severe acute respiratory syndrome coronavirus 2 (SARS-CoV-2) was first identified in Wuhan (China) as the cause of a novel viral pneumonia [14]. Later, the World Health Organization (WHO) announced that SARS-CoV-2 is the causative agent of COVID-19, and in March 2020 the WHO declared COVID-19 a pandemic [15]. This pandemic has had an unprecedented social and economic impact on the lives of billions of people worldwide, having caused over 3.8 million deaths and more than 178 million cases worldwide as of 22 June 2021 [16].

As a first step in the pathogenesis process, SARS-CoV-2 uses the angiotensin-converting enzyme 2 (ACE2) receptor for entry and the transmembrane serine protease 2 (TMPRSS2) for spike protein priming in host cells [17]. As a result of infection, patients have reported symptoms varying from muscle pain, loss of the sense of smell and taste, shortness of breath, to more severe manifestations such as ARDS, cardiovascular abnormalities including cardiomyopathy, carditis, arrhythmia, and heart failure which are linked to the robust cytokine storm in infected patients [18,19]. Long-term manifestations of the disease have also been reported in recovered patients [20]. The prognosis of COVID-19 has been linked to the health history and underlying conditions of infected individuals, among which smoking has been proposed to render patients more likely to become infected and develop more severe symptoms of the disease [13,21,22,23,24].

Studies have shown that the lung tissue of current smokers [13,22,23,24] and former smokers (cessation less than 10 years) [25] presents higher levels of ACE2 compared with non-smokers. One could speculate that the mouth is one of the primary entry sites for the virus before it reaches the lower respiratory tract, as early symptoms of infections are characterized, among others, by dry mouth, distortion of the perception of taste (dysgeusia) or complete loss of taste (ageusia) [26]. So far, few studies have shown the presence of SARS-CoV-2 receptors in the epithelial cells of the oral mucosa [27,28,29]. Other studies have shown that SARS-COV-2 is found in the dental biofilm of COVID-19 patients [30], and that SARS-CoV-2 can infect cells in the oral cavity and saliva [31]. However, there are no studies showing the effects of cigarette smoke on SARS-CoV-2 infection in the oral cavity. Therefore, we hypothesized that cigarette smoke could increase the expression of SARS-CoV-2 receptors, which would favor SARS-CoV-2 infection in human gingival epithelial cells.

## 2. Results

### 2.1. Cigarette Smoke Condensates Enhance ACE2 and TMPRSS2 Expression in Gingival Epithelial Cells

**Figure 1:**
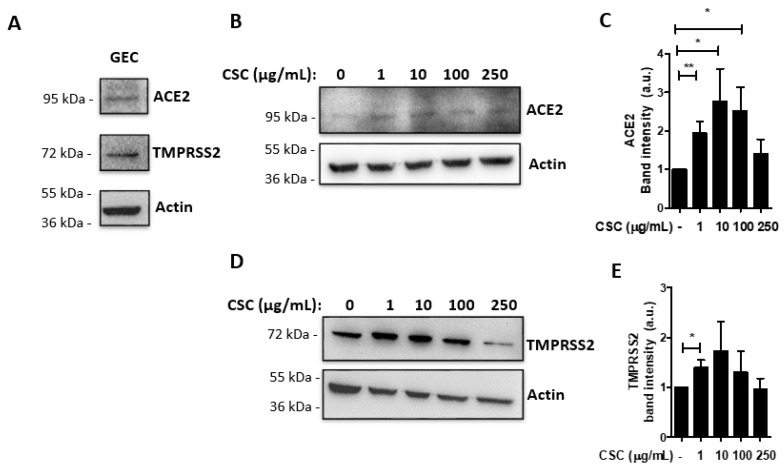
Cigarette smoke condensate treatment increases angiotensin-converting enzyme 2 (ACE2) and transmembrane serine protease 2 (TMPRSS2) levels in human gingival epithelial cells. Human gingival epithelial cells (GECs) were treated with or without cigarette smoke condensates (CSC) at different concentrations for 24 h, and expression of ACE2 and TMPRSS2 were determined by Western blot. (**A**) shows a representative Western blot image for ACE2 and TMPRSS2 in untreated GECs. (**B**) shows representative Western blot images for ACE2 and (**D**) TMPRSS2 after treatment with CSC at different concentrations for 24 h. (**C**,**E**) Graphs show average ± SEM of band intensity (arbitrary units) for ACE2 (**C**) and TMPRSS2 (**E**)**.** (**A**) *n* = 3, (**B**,**C**) *n* = 7, (**D**,**E**) *n* = 6. * *p* ≤ 0.05; ** *p* ≤ 0.01.

We initially examined whether receptors for SARS-CoV-2 infection are expressed in human oral cells. We thus performed Western blots of untreated human gingival epithelial cells (GECs) and observed expression of ACE2 and TMPRSS2 at the protein level (Figure 1A). We then examined whether treatment with cigarette smoke condensates (CSC) at different concentrations [32] could modulate the expression of these proteins. After 24 h of CSC treatment, we observed that the CSC concentration of 1 μg/mL induced a 2-fold increase of ACE2 expression, and that 10 μg/mL or 100 μg/mL led to approximately a 2.5-fold increase, compared with untreated cells (Figure 1B,C). On the other hand, CSC had a less prominent, but still significant, effect on TMPRSS2 expression after CSC treatment at 1 μg/mL. To investigate whether the concentrations of CSC used in this study were cytotoxic, we quantified LDH release by GECs after 24 h of CSC treatment. Cell death was not detectable in our experiments after CSC treatment (Figure S1). These results confirm that GECs express SARS-CoV-2 receptors, which are upregulated after CSC treatment.

### 2.2. Activation of the Arzyl Hydrocarbon Receptor Upregulates ACE2 Expression in Gingival Epithelial Cells

Molecules from cigarette smoke can stimulate the aryl hydrocarbon receptor (AhR). AhR is an ubiquitously-expressed transcription factor that is activated by a variety of agonists, including molecules found in cigarette smoke [33]. A xenobiont, 2,3,7,8-tetrachlorodibenzo-p-dioxin (TCDD), is the best known AhR ligand [33,34]. AhR activation by TCDD can lead to pathological effects, while AhR activation by other ligands, such as bacterial products, can be protective [33].

**Figure 2:**
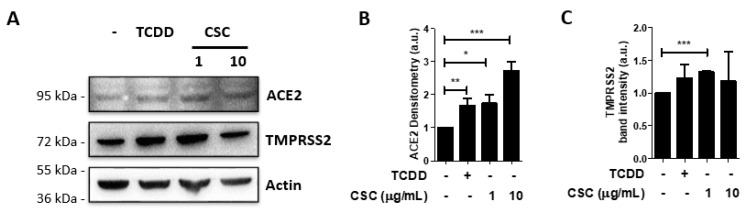
2,3,7,8-tetrachlorodibenzo-p-dioxin (TCDD) treatment increases angiotensin-converting enzyme 2 (ACE2) levels, but not transmembrane serine protease 2 (TMPRSS2), in human gingival epithelial cells. GECs were treated with or without cigarette smoke condensates (CSC) at different concentrations as indicated, or with TCDD (10 nM), for 24 h. Expression of ACE2 and TMPRSS2 were determined by Western blot. (**A**) shows representative Western blots for ACE2 and TMPRSS2 in GECs after treatment with or without CSC or TCDD. (**B**,**C**) Graphs show average ± SEM of band intensity (arbitrary units) for ACE2 (**B**) and TMPRSS2 (**C**)**.** Data shown are representative of at least 3 independent experiments. * *p* ≤ 0.05; ** *p* ≤ 0.01; *** *p* ≤ 0.001.

We hypothesized that AhR could be involved in recognition of CSC by GECs, and that activation of this receptor could lead to an increase in ACE2 and TMPRSS2 expression upon CSC treatment. We therefore examined the expression of ACE2 and TMPRSS2 by Western blot in GECs treated with TCDD or CSC for 24 h. We observed that TCDD treatment increased ACE2 expression in GECs to levels similar to those obtained by treatment with CSC at 1 μg/mL (Figure 2A,B). We also showed that the treatment with CSC at 10 μg/mL induced even higher levels of ACE2 (Figure 2A,B). However, even though TMPRSS2 expression levels increased after treatment with CSC 1 μg/mL, treatment with neither TCDD nor CSC at 10 μg/mL significantly increased the expression of the protease. These data suggest that AhR activation is involved in the increase of ACE2, but not TMPRSS2, in GECs.

### 2.3. AhR Is Expressed and Activated in Gingival Epithelial Cells by Cigarette Smoke Condensate Treatment

**Figure 3:**
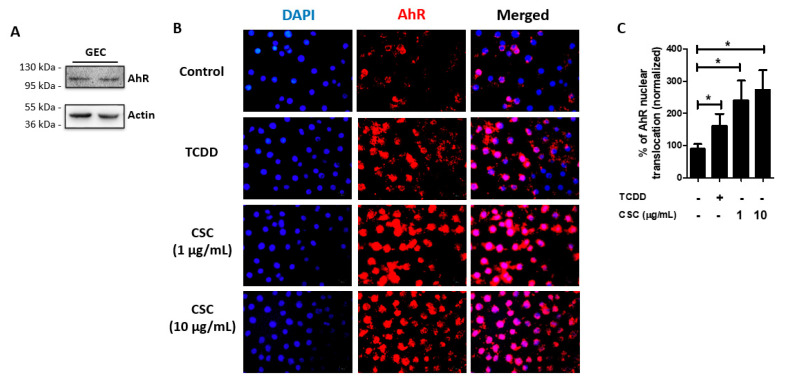
Aryl hydrocarbon receptor (AhR) is activated by treatment with cigarette smoke condensate in gingival epithelial cells. Human gingival epithelial cells (GECs) were treated with or without cigarette smoke condensates (CSC) at different concentrations as indicated, or with 2,3,7,8-tetrachlorodibenzo-p-dioxin (TCDD, 10 nM), for 24 h. AhR expression and activation was determined by Western blot and immunofluorescence microscopy, respectively. (**A**) shows a representative Western blot for AhR in unstimulated GECs. (**B**) shows representative images of immunofluorescence for AhR. (**C**) Graph shows average percentage ± SEM of AhR translocation to the nucleus by measuring the intensity of fluorescence using Image J. (**A–C**) *n* = 3. * *p* ≤ 0.05.

Next, we examined whether AhR is expressed in GECs in order to determine whether AhR intracellular signaling could be involved in GEC recognition of CSC. AhR is widely expressed in the human body and in particular in the immune system [34], but its expression in GECs needed to be confirmed. We therefore demonstrated by Western blot that AhR is expressed in unstimulated GECs (Figure 3A).

AhR is a cytoplasmic receptor that translocates to the nucleus upon activation [33,34,35]. Thus, we evaluated whether AhR would be activated by CSC treatment, using TCDD as a positive control. We therefore treated GECs for 1 h with or without TCDD (10 nM) or CSC (1 or 10 μg/mL) and visualized AhR translocation to the nucleus by immunofluorescence. We observed that TCDD induced AhR nuclear translocation, and that treatment with CSC at 1 or 10 μg/mL could also induce nuclear translocation of AhR (Figure 3B). Quantification of the images in Figure 3B showed the percentage of AhR nuclear translocation compared with the control group (Figure 3C). Our results thus confirm a previous study that oral keratinocytes express functional AhR [36], and reveal that AhR is activated by CSC treatment.

### 2.4. Cigarette Smoke Condensates Enhance SARS-CoV-2 Pseudovirus Internalization in Gingival Epithelial Cells

**Figure 4:**
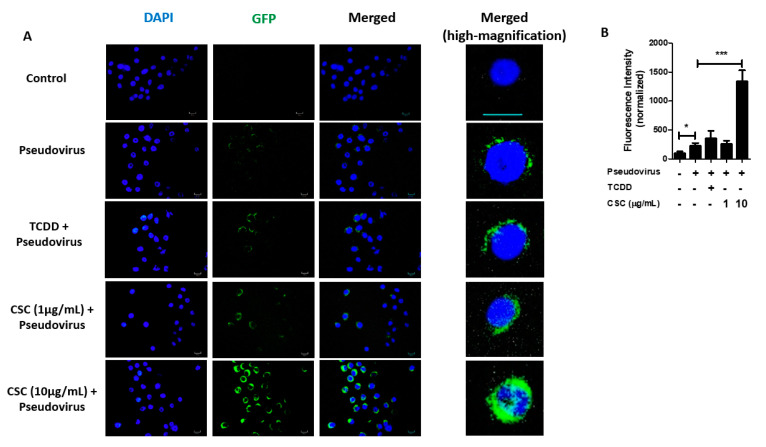
Pretreatment with cigarette smoke condensates increases severe acute respiratory syndrome coronavirus 2 (SARS-CoV-2) pseudovirus infection in gingival epithelial cells. Human gingival epithelial cells (GECs) were treated with or without cigarette smoke condensates at different concentrations as indicated, or with 2,3,7,8-tetrachlorodibenzo-p-dioxin (TCDD, 10 nM), for 24 h, prior to infection with SARS-CoV-2 pseudotyped GFP-tagged lentivirus for an additional 24 h. SARS-CoV-2 pseudovirus infection was visualized by immunofluorescence microscopy. (**A**) shows representative images of immunofluorescence for GFP-tagged SARS-CoV-2 pseudovirus. (**B**) Graph shows average percentage ± SEM of GFP fluorescence intensity, measured using Image J. (**A**,**B**) *n* = 3. * *p* ≤ 0.05; *** *p* ≤ 0.001. Diameter bar = 100 μm.

A recent study has shown that SARS-CoV-2 can infect cells in the human oral cavity and saliva [31]. Given that ACE2 mediates SARS-COV-2 infection and that ACE2 expression was significantly increased in GECs after treatment with TCDD or CSC, we hypothesized that the treatment with these compounds might favor SARS-CoV-2 infection in GECs. Thus, we pretreated GECs with or without TCDD (10 nM) or CSC (1 or 10 μg/mL) for 24 h before infecting the cells with SARS-CoV-2 GFP-tagged pseudovirus for an additional 24 h. We then measured viral infection by immunofluorescence microscopy. We confirmed that SARS-CoV-2 pseudovirus could infect human GECs, whether or not the cells had been pretreated (Figure 4A). GECs pretreated with TCDD or CSC at 1 μg/mL did not show a change in SARS-CoV-2 pseudovirus internalization compared with infection of untreated GECs (Figure 4A). However, GECs pretreated with CSC at 10 μg/mL showed a significant increase in SARS-CoV-2 pseudovirus infection compared with infection of untreated control GECs (Figure 4A). Quantification of SARS-CoV-2 pseudovirus internalization levels is shown in Figure 4B. Thus, our data suggest that CSC treatment increases the levels of ACE2 in GECs, which enhances SARS-CoV-2 pseudovirus internalization.

### 2.5. Cigarette Smoke Condensates Promote SARS-CoV-2 Pseudovirus Internalization via AhR Activation in Gingival Epithelial Cells

**Figure 5:**
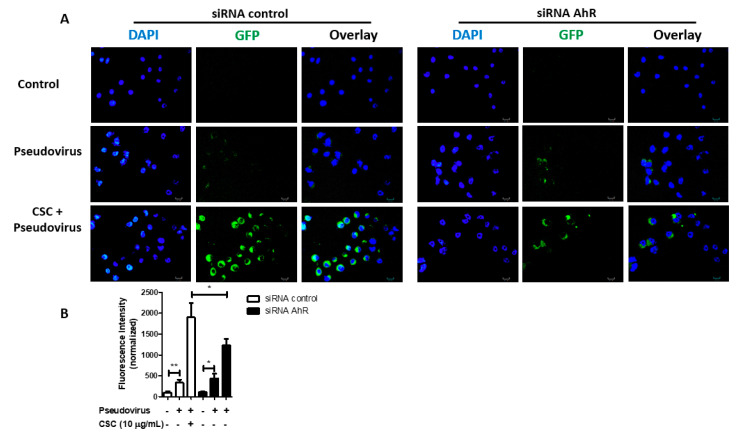
Treatment with cigarette smoke condensates increases severe acute respiratory syndrome coronavirus 2 (SARS-CoV-2) pseudovirus infection in gingival epithelial cells via aryl hydrocarbon receptor (AhR) signaling. Human gingival epithelial cells (GECs) were transfected with siRNA sequences against AhR or an unrelated target, and treated with or without cigarette smoke condensate (10 μg/mL) for 24 h, prior to infection with SARS-CoV-2 pseudotyped GFP-tagged lentivirus for an additional 24 h. SARS-CoV-2 pseudotyped GFP-tagged lentivirus infection was visualized by immunofluorescence microscopy. (**A**) shows representative images of immunofluorescence for GFP-tagged SARS-CoV-2 pseudovirus. (**B**) Graph shows average percentage ± SEM of GFP fluorescence intensity, measured using Image J. (**A**,**B**) *n* = 3. * *p* ≤ 0.05; ** *p* ≤ 0.01; Diameter bar = 100 μm.

**Figure 6:**
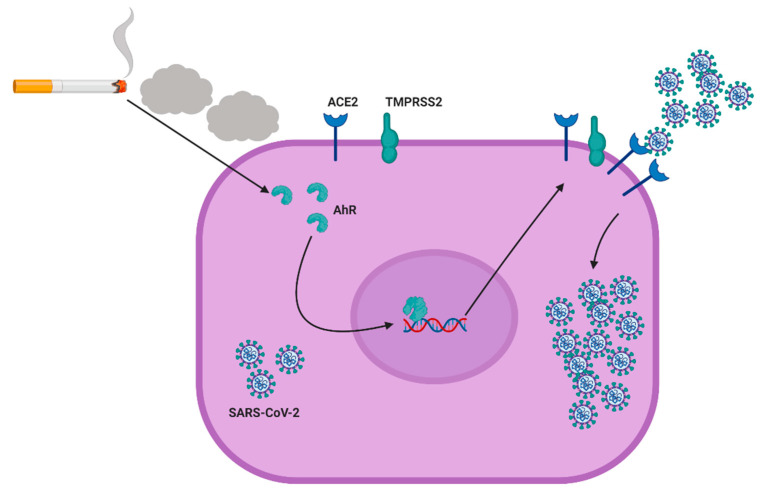
Proposed scheme for the mechanism involved in cigarette smoke-induced enhancement of severe acute respiratory syndrome coronavirus 2 (SARS-CoV-2) infection in human gingival epithelial cells. Cigarette smoke activates aryl hydrocarbon receptor (AhR) in human gingival epithelial cells, which increases the surface levels of angiotensin-converting enzyme 2 (ACE2). Increased expression of ACE2 facilitates SARS-CoV-2 infection of the oral epithelial cells.

Since CSC induces AhR activation in GECs (Figure 3) and AhR activation increases ACE2 expression in these cells (Figure 2), we tested whether CSC potentiated SARS-CoV-2 pseudovirus infection in GECs via AhR activation. We used siRNA to deplete AhR in GECs before treating the cells with TCDD or CSC, and then infecting the cells with SARS-CoV-2 pseudovirus. We could successfully deplete AhR expression in GECs after the siRNA treatment, as shown by Western blot (Supplementary Figure S2, Figure 2). Using cells that were transfected with siRNA control, we observed high levels of SARS-CoV-2 pseudovirus internalization after CSC treatment (Figure 5A). GECs transfected with siRNA against AhR and treated with CSC showed lower levels of SARS-CoV-2 pseudovirus internalization, compared with GECs transfected with siRNA control and treated with CSC. Figure 5B shows the quantification of the infection observed in Figure 5A. Taken together, the data show that CSC treatment activated AhR signaling in GECs, and thereby increased expression of the SARS-CoV-2 receptor ACE2. Increased levels of ACE2 favored SARS-CoV-2 pseudovirus infection in an AhR-signaling dependent manner (Figure 6).

## 3. Discussion

Cigarette smoke is harmful [1] for oral [2,8,37] and systemic health [2,5,38,39] in humans. A systematic review and meta-analysis of 16 articles describing 11,322 COVID-19 patients showed a link between history of smoking and severe cases of COVID-19 [13]. This meta-analysis study showed that active smokers were twice as likely to develop severe COVID-19, compared with non-smokers [13]. Cigarette smoke can impact human immune responses and is linked to several clinical conditions, which could contribute to a higher susceptibility to COVID-19 [40,41,42]. The current study suggests a mechanism whereby cigarette smoke may increase the risk of SARS-CoV-2 infection in GECs (Figure 6). Our data suggest that smokers could present AhR-dependent upregulation of ACE2 in the oral cavity, which could increase susceptibility to SARS-CoV-2 infection, and therefore increase the risk of contracting severe symptoms of COVID-19.

Our data (Figure S1) confirms a previous study which demonstrated that CSC at 250 μg/mL is not cytotoxic for GECs [32]. In fact, CSC at 250 μg/mL is equivalent to 6 μg/mL of nicotine [32,43]. Because the concentration of nicotine in the saliva of smokers ranges from 0.9 to 4.6 μg/mL [43,44], we considered concentrations of CSC lower than 250 μg/mL to be physiologically relevant. Therefore, in our study, the physiologically-relevant concentration of CSC (e.g., 10 μg/mL) upregulated ACE2 and stimulated SARS-CoV-2 internalization in GECs.

Consistent with our observation that CSC increases ACE2 expression in human oral epithelial cells, other studies have shown that cigarette smoke increases ACE2 expression in lung tissues [22,23,24]. In fact, a recent study showed that ACE2 expression was significantly increased in bronchial epithelial cells in current smokers and former smokers, and was significantly correlated with pack-years of smoking [25]. A meta-analysis showed that the *ACE2* and *TMPRSS2* genes are widely expressed in human tissues where they are induced under pro-inflammatory conditions [45]. Interestingly, another study showed that particulate matter upregulates ACE2 expression and exacerbates susceptibility to SARS-CoV-2 infection in lung tissue of humanized ACE2 mice [46]. Given that ACE2 mediates SARS-CoV-2 infection in host cells, we also demonstrated that CSC enhances SARS-CoV-2 pseudovirus infection in GECs. The effects of CSC on ACE2 expression in oral cells suggests the possibility of therapeutic intervention, since ACE2 antibodies or soluble recombinant ACE2 can attenuate viral entry and infection by SARS-CoV-2 in other cells [17,47].

Our data are also consistent with a recent study showing that kynurenine (a commonly-used endogenous ligand) activates AhR, which binds to the promoter of the *ACE2* gene and upregulates ACE2 expression in the BEAS-2B lung cell line, as well as in lung tissue of mice and macaques [48]. The same study also showed that macaques infected with SARS-CoV-2 and treated with AhR inhibitor presented decreased ACE2 expression, and ameliorated pathological damage, compared with the infected but untreated group [48].

Our results show that CSC modulates ACE2 expression and thus, increases SARS-CoV-2 infection via activation of AhR. Although TMPRSS2 expression increased after exposure to CSC in GECs, we did not observe a change in the expression of the protease after AhR activation using TCDD. This suggests that TMPRSS2 may be regulated via a different pathway than for ACE2. In this context, it has been reported that smoking increased androgen levels [49], and it has been proposed that TMPRSS2 expression is modulated by the androgen pathway [50,51]. We therefore speculate that TMPRSS2 expression in GECs may be partially regulated through AhR-independent pathways.

Several risk factors for severe symptoms of COVID-19 have been identified, including old age, male sex, underlying comorbidities such as hypertension, diabetes, obesity, chronic lung diseases, heart, liver, and kidney diseases, cancer, and immunodeficiencies [52]. Interestingly, smoking has been linked to several of these comorbidities, such as hypertension [4], cancer [6,7], and diabetes [9]. Future epidemiological and clinical studies should also investigate whether cigarette smoke could contribute to COVID-19 susceptibility and severity by affecting comorbidities shared with COVID-19.

Unfortunately, smoking is a very difficult addiction to break, even for those who want to quit [53]. Quitting smoking is beneficial to the overall health at any age, and cigarette smokers who quit before age 35 have mortality rates that are similar to those who never smoked [54,55]. Since 2002, the number of people who quit smoking has been increasing, and studies suggest that mass media campaigns, increases in the price of tobacco products, and smoke-free policies contribute to this trend [55]. In the present study, we show how cigarette smoke could increase sensitivity to SARS-CoV-2 infection in oral cells. Thus, besides all the beneficial effects on people’s health, we suggest that smoking cessation could be advantageous for reducing the susceptibility of coronavirus infection of the oral cavity.

Other studies have shown that oral cells might serve as a route of coronavirus infection or transmission from the oral cavity to the lower respiratory tract [27,28,31]. Since SARS-CoV-2 could also be transmitted to other individuals through activities involving the oral cavity such as speaking, breathing, coughing, sneezing and even singing [56,57], the infection of oral cells could be a source or route for extra-oral transmission or coronavirus to other individuals. Thus, understanding the mechanisms involved in SARS-CoV- 2 infection in cells of the oral cavity may suggest strategies for inhibiting both infection in the host and transmission to other individuals. Epidemiologic and clinical studies are needed to confirm our hypothesis that smoking increases SARS-CoV-2 infection susceptibility in the oral cavity, and that AhR signaling could be a potential target for treatment and prevention of coronavirus infection and transmission.

## 4. Materials and Methods

### 4.1. Human Gingival Epithelial Cells and Cell Culture 

Immortalized human oral gingival keratinocytes, IHGK (HPV-16GM), were purchased from Applied Biological Materials (ABM, cat# T0717, Richmond, CA, USA), and maintained as we previously described [58]. Briefly, gingival epithelial cells (GECs) were cultured and maintained in Keratinocyte serum-free medium (KSFM, Gibco, Gaithersburg, MO, USA) suplemented with 30 μg/mL of bovine pituitary extract (Gibco), 0.2 ng/mL of human recombinant epidermal growth factor (Gibco), 100 U/mL of penicillin and 100 μg/mL of streptomycin (Gibco). All cells were maintained at 37 °C in a humidified incubator containing 5% CO_2_. Cell counts were performed using Trypan Blue (Sigma-Aldrich, MO, USA) exclusion for seeding only viable cells for the experiments. 

### 4.2. Reagents 

Cigarette smoke condensates (CSC) were obtained from Murty Pharmaceuticals, Inc. (Lexington, KY, USA). Tetrachlorodibenzo-p-dioxin solution (TCDD, cat #48599), complete protease inhibitor cocktail (cat# 11836145001) were from Millipore Sigma (St. Loius, MO, USA). 5X RIPA Lysis buffer was from (Milipore, Temecula, CA, USA). Lactate dehydrogenase (LDH) detection assay kit (cat# C20300) was from Thermofisher (Waltham, MA, USA). Human ACE2 policlonal antibody (cat# AF933) was from R&D Systems (Minneapolis, MN, USA). TMPRSS2 (cat# sc-515727), AhR (cat# sc-133088), m-IgGk Bp-PE (cat# sc-516141) were from Santa Cruz Biotechnology (Santa Cruz, CA, USA). Secondary antibodies goat anti-mouse (cat# ab9700) and rabbit anti-goat (cat# ab97023) were from Abcam (Cambridge, MA, USA). OPTIMEM was from Gibco (USA).

### 4.3. SARS-CoV-2 Spike Protein Pseudotyped GFP Lentivirus 

SARS-CoV-2 Spike protein pseudotyped GFP lentivirus expressing SARS-CoV-2 Spike protein (SARS-CoV-2 pseudovirus) was obtained from Creative Biogene (CoV-001, Shirley, NY, USA). The SARS-CoV-2 pseudovirus is GFP-tagged and was used for imaging analysis.

For quantification of the SARS-CoV-2 pseudovirus internalization, GECs (1 × 10^4^ cells/well) were seeded on 18 mm coveslips in 24-well plates with OPTIMEM and were incubated overnight. On day 1, cells were treated with or without CSC at a final concentration of 1 or 10 μg/mL, or with TCDD at 10 nM in OPTIMEM for 24 h. On day 2, SARS-CoV-2 pseudovirus was added to achieve the multiplicity of infection (MOI) of 20. On day 3, cells were washed twice with warm and sterile PBS, and mounted on slides using Vectashield^®^ Hardset™ Antifade Mounting Medium with DAPI (Vector Laboratories, Burlingame, CA, USA). Images were acquired using a Nikon Eclipse 50i fluorescence microscope with an Infinity 3 camera and Lumenera Infinity Analyze 6.3 software. ImageJ was used to quantify internalization of SARS-CoV-2 pseudovirus by measuring GFP intensity of fluorescence

### 4.4. Transient RNA Depletion Using siRNA

Expression of AhR in GECs was repressed using Silencer^®^ Select siRNA (cat#4390824, siRNA ID s1199) from Ambion by Life Technologies. A negative control transfection was performed using Silencer^®^ Select Negative Control #1 siRNA (cat# 4390843). Briefly, cells were treated with 50 nM [59] of the appropriate Silencer^®^ Select siRNA using lipofectamine 2000 (Invitrogen, Carlsbad, CA, USA) in OPTIMEM, following manufacturer’s instructions. After 24 h of siRNA transfection, knockdown efficiency and specificity was confirmed by Western blot (Figure S2), as described in the Western blot section.

For experiments using AhR transient knockdown GECs to examine SARS-CoV-2 pseudovirus infection, GECs were seeded on coverslips in 24-well plates, before being transfected for 24 h, as described above. After AhR depletion, GECs were infected with SARS-CoV-2 pseudovirus at an MOI of 20 for additional 24 h. Infection was detected and quantified as described in the section above.

### 4.5. Werstern Blot Analysis

For the Western blot experiments, 2 × 10^5^ GECs were seeded on 6-well plates (Corning) and incubated at 37 °C, 5% CO_2_ for 24 h to achieve around 75–80% of confluence. On day 1, cells were treated or not with CSC at different concentrations, or with TCDD for 24 h, as indicated in the experiments. Cellular extracts were collected in lysis buffer supplemented with complete protease inhibitor cocktail. Lysates were collected for Western blot analysis to measure ACE2 (100 kDa), TMPRSS2 (70 kDa) or AhR (122 kDa) expression levels.

Cell lysates were collected as previously described [58]. Briefly, cell lysates were collected in ice chilled lysis buffer supplemented with 1% protease inhibitor cocktail. Protein concentration was measured using BCA assay (Thermofisher) according to manufacturer’s instructions. Then, 20 μg of proteins in reducing sample buffer (Thermofisher) were denatured at 96 °C for 5 min and electropheretically resolved on 12% SDS-PAGE. Separated proteins were transfered to a polyvinylidene difluoride membrane (PVDF membranes, EMD Millipore, Darmstadt, Germany) via a wet electroblotting system (Bio-Rad, Hercules, CA, USA). The membranes were blocked with 5% skim milk in Tween-20 TBS buffer (TBST) and then probed with respective antibodies: Polyclonal ACE2 antibody (1:2000) with secondary antibody Goat anti-rabbit (1:60,000) (Abcam). Monoclonal TMPRSS2 antibody (1:1000) (Santa Cruz Biotechnology) with Secondary antibody Goat anti-mouse (1:15,000) (Milipore). Monoclonal AhR (1:2000) with Secondary antibody Goat anti-mouse (1:15,000) (Milipore). All primary antibodies probing was incubated overnight while secondary antibody incubation was done at room temperature for 1h. Membranes were washed 3 times for 5 min after each antibody incubation and visualised using immobulin Forte western HRP substrate (Millipore) and Chemidoc imaging system’s image lab software Version 6.0.1 build 34 (Bio-Rad). Images were quantified by ImageJ analysis by measuring band intensities. Data was normalized to control values.

### 4.6. AhR Immunofluorescence

GECs (1 × 10^4^) were seeded on 18 mm coverslips in 24-well plate (Corning) to achieve around 80% of confluence, followed by treatment with or without CSC at 1 or 10 μg/mL, or the AhR agonist TCDD at 10 nM. Cells were incubated for 1 h at 37 °C, 5% CO_2_, and then washed and fixed with cold methanol at room temperature 10 min. After 3 washes with PBS, overnight incubation for permeabilization and blocking was performed with a solution of 0.2% Triton X-100 (Sigma-Aldrich) in 5% Goat Serum (Sigma-Aldrich) and 1X PBS (Gibco). Subsequently, cells were probed with mouse monoclonal antibody, anti-AhR at a concentration of 1:50 prepared in 0.05% Triton X-100 in 5% Goat Serum/PBS with overnight incubation at 4 °C, in agitation. After 3 washes with PBS, Goat anti-mouse m- IgGk Bp-PE prepared in 0.05% Triton X-100/5% goat Serum/ PBS at 1:200 was added to the wells and incubated at room temperature for 2 h, protected from light. Cells were washed three times, counterstained, and mounted on a slide using Vectashield^®^ Hardset™ Antifade Mounting Medium with DAPI. Images were acquired using a Nikon Eclipse 50i fluorescence microscope with an Infinity 3 camera and the Lumenera Infinity Analyze 6.3 software. AhR nuclear translocation was quantified using Image J software, following the protocol by Wessel and Hanson [60]. Data was normalized to control values.

### 4.7. Lactate Dehydrogenase Detection Assay 

To measure cell viability, LDH levels were measured spectrophotometrically using CyQUANT LDH Cytotoxicity Assay kit (Thermofisher, Waltham, MA, USA). GECs were seeded overnight on a 24-well plate (Corning), followed by treatments with or without CSC at different concentrations (1, 10, 100, 250 μg/mL) for 24 h. Lysis buffer or water was added during the last 40 min, and served as internal controls. After sample collection, supernatants were transfered to clear flat-bottom 96-well plates (Corning), followed by LDH substrate addition and incubation for 30 min at room temperature, profected from light. Prior to measuring absorbance, the reaction was quenched using the Stop Solution from the kit. Absorbance values were recorded at 490 nm and 680 nm using the Versamax Tunable Microplate Reader (Molecular Devices, Ramsey, MN, USA). Cells treated with lysis buffer were used as positive control and defined as 100% of cell death, cells treated with water were used to provide spontaneous cell death, and cells with no treatment were used as negative controls in the experiments. In order to obtain the percentage of cytotoxicity, we subtracted the readings at 680 nm from the readings at 490 nm, and values were then submitted to the following equation provided by the manufacturer:

$$\%\mathrm{Cytotoxicity}=\frac{(\mathrm{Compound-treated}\;\mathrm{LDH}\;\mathrm{activity}\;-\;\mathrm{Spontaneous}\;\mathrm{LDH}\;\mathrm{activity})\;\times \;100}{\left(\mathrm{Maximum}\;\mathrm{LDH}\;\mathrm{activity}\;-\;\mathrm{Spontaneous}\;\mathrm{LDH}\;\mathrm{activity}\right)}$$

### 4.8. Statistical Analysis 

Western blots and fluorescence microscopy data were quantitatively analyzed using ImageJ software. Statistics comparing two groups were carried out using one-tailed *t*-test performed using Graph Pad Prism v.5 software. Statistical differences were shown as asterisks, where: *** *p* < 0.001, ** *p* < 0.01, * *p* < 0.05.

## Data Availability

The data presented in this study are available in either this article or in the Supplementary Material.

## Supplementary Materials

The following are available online at https://www.mdpi.com/article/10.3390/ijms22147669/s1, Figure S1: Treatment with cigarette smoke condensates for 24 h does not induce cell death of human gingival epithelial cells., Figure S2: Small interference RNA against AhR decreases expression in human gingival epithelial cells (GECs).
