# Supplementary material for: Cigarette Smoke Stimulates SARS-CoV-2 Internalization by Activating AhR and Increasing ACE2 Expression in Human Gingival Epithelial Cells
Source: Int J Mol Sci. 2021 Jul 18;22(14):7669. doi: 10.3390/ijms22147669 (PMC8307094; doi:10.3390/ijms22147669)
Supplement: Supplementary file 1 [file ijms-22-07669-s001.zip › ijms-1292624-SI.pdf]

Supplementary material for the study entitled “Cigarette smoke stimulates SARS-CoV-2 internalization by activation AhR and increasing ACE2 expression in human gingival epithelial cells” by Almeida-da-Silva et al. 2021.

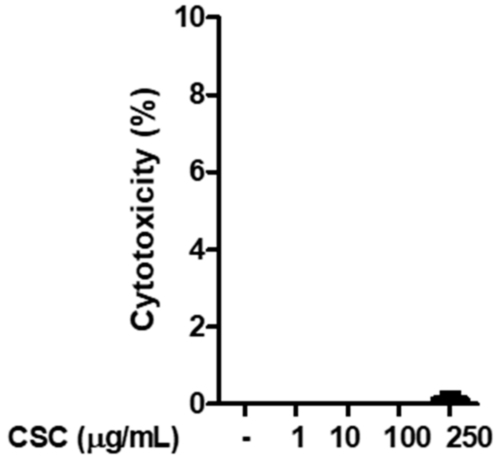

Figure S1. Treatment with cigarette smoke condensates for 24 h does not induce cell death of human gingival epithelial cells (GECs). Cell death was analyzed lactate dehydrogenase (LDH) release. Lysis buffer provided in the kit served as positive control and was defined as 100% of cell death. Experimental values were then analyzed following manufacturer’s instructions as detailed in Materials and Methods (4.7 Lactate dehydrogenase detection assay). Graph shows the percentage of cytotoxicity (mean ± SEM) induced by treatment of GECs with different concentrations of CSC (as indicated) for 24 h. Graph is representative of 4 independent experiments.

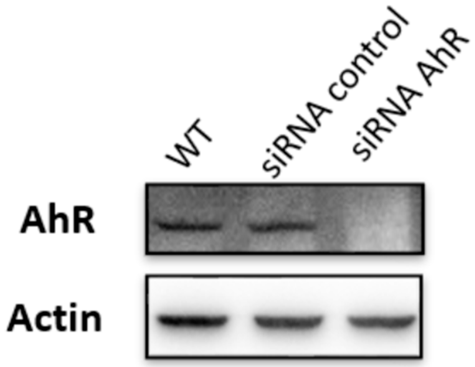

Figure S2. Small interference RNA against aryl hydrocarbon receptor (AhR) decreases expression in human gingival epithelial cells (GECs). The cells were transfected with siRNA sequences against AhR or against an unrelated sequence for 24 h, prior to collection of cell extracts. AhR expression was determined by Western blot. Image shows Western blot for AhR. Image is representative of 3 independent experiments.
